# Supplementary material for: Association between ‘Emergency obstetric & newborn care readiness’ and delivery service utilization in Bangladesh: Evidence from national health facility assessment surveys
Source: PLoS One. 2025 May 27;20(5):e0297734. doi: 10.1371/journal.pone.0297734 (PMC12112276; doi:10.1371/journal.pone.0297734)
Supplement: Attachment — Submitted filename: Reviewer comments_20240226.docx [file pone.0297734.s001.docx]

**Reviewer comments**

The authors have made commendable effort to make sense of available secondary data to show EmONC QoC status in Bangladesh.

**The manuscript requires major revisions**

1. The title is too broad and misleading. First as the study is focused on EmONC which is an intervention designed to improve maternal and newborn health, the title should reflect it as such.
2. **Abstract section**

Background/introduction is missing

Objectives do not reflect the findings of the study

Methods : Indicate the number of surveyed health facilities in 2014 and 2017 and those providing basic EmONC and Comprehensive

Results :

- One of the objectives of your study is to assess service utilization but not shown under the result section
- Present the figures in proportion/percentage in addition to the #, to make it more informative
- Please stratify the findings by basic and comprehensive EmONC
- Conclusion : Without showing the proportion the given information/data are not enough to conclude that there is improvement. You need to show whether there were statistically significant change in the part you presented the results to claim that there are improvement in 2017 compared to 2017.

The conclusion has gone far from what you have shown under the results

3. **Background/Introduction section**

- It lacks logical flow
- It requires high level of analysis and removing redundant ideas
- The objective of the manuscripts you have it here is different from the one in the abstract section. It should be reconciled. The one you have it here reflect your study better.

4. **Methods section**

Please make it clear which facilities are providing basic EmONC and comprehensive EmONC. Without having this information, it is difficult to review the paper. Please clearly describe the standards for Emergency Obstetrics and Neonatal Care (EmONC) recommended by WHO and contextualization of the Bangladeshi system . Eg

| EmONC standard indicators | Basic EmoNC facility | Comprehensive EmONC Facility |
| --- | --- | --- |
| Population served | 25,000? | 100,000? |
| Availability per district |  |  |
| Signal functions | 7 | 9 |
| Health care workers |  |  |
| Supplies and pharmaceuticals |  |  |
| Infrastructure |  |  |
| # of facilities surveyed in 2014 (SPA) |  |  |
| # of facilities surveyed in 2017 (SPA |  |  |

In the SPA survey, how many of the 140 and 141 facilities surveyed in 2014 and 2017 respectively were basic EmONC, comprehensive EmONC and non-EmONC (Be remined those facilities not providing either the 7 or the 9 signal functions are non-EmONC, which may be excluded from the analysis.

All the analysis should be stratified by Basic and comprehensive. If you wish to present only the comprehensive EmONC, you can do so but clearly indicate in your objective, methods and background.

Although EmoNC has clear standard on the type of infrastructure, supplies, medicine, manpower and services provided, your manuscript gave emphasis on C. Section and required resources for it. 1) It is not clear why only C. Section was given emphasis in the first place, 2) by focusing on C. Section, the manuscript could limit its relevance for few area experts.

Are the health facilities surveyed in 2014 and 2017 the same? Are they like cohorts? Often there are some facilities to be added or removed when having cross-sectional SPA survey in different places.

It is not clear what do you mean by “the 2017 SPA survey was conducted in 2019”. Did you mean the 2017 SPA reports were published, in 2019 ?

As you described, Donabedian is a comprehensive model that enable to assess structure, process and outcomes. However, in your study when calculating the QoC index you looked into the availability of signal functions, (only the process). By doing so you diverged from the model assumption/premises, which is not clear. Make it clear why you chose this model and how it guided your study. I would suggest you to have a good understanding of the model, QoC and the EmONC concepts and applications, then it would be easy for you to be guided by the model for assessing EmONC care quality.

The description under the delivery service utilization sub-title should be described under variable and measurement section not under the analysis. Moreover, the facility categorization you are referring to is made to show how repeated exposure/practice increase skill, care quality and hence health outcomes. If you are applying this, you should also categorize the facilities/SPA data accordingly based on the volume of care they provide taking into account the national standards.

In EmONC, the signal function related to neonatal care is “ Perform basic neonatal resuscitation”. You have Kangaroo Mother Care (KMC), which is not EmONC signal function but can be an additional variable but you should have a variable to measure a signal function for newborn care as recommended by the EmONC standard.

On the part about the availability of midwife/nurse, indicate how you quantified/ classified as per the EmONC standard under variables and measurement section eg; for the # of deliveries the facility conducts how many midwives/nurses are there. Are they below or above the expected standard as these would have significant implication on the QoC and to show the health facility readiness?

**Result section**

You have some information in the results section, which should be part of the method section. Eg: about the analyzed samples, *" Out of 141 facilities in 2017, a total of 7 facilities had missing information and finally 134 facilities (141-7=134 facilities) were included in the service utilization analysis"*

Why death review is highlighted in the first paragraph of all the maternal and newborn health indicators? There is no mention in the background or method section related to it.

When comparing 2014 with 2017 data and other comparisons, test of significance is important to show whether there are changes between the two survey points.

In table 2, you have listed all the nine-signal function for comprehensive EmONC. From the figure, I can see few of the facilities (around one fourth of the surveyed facilities were providing comprehensive EmONC services). Here, the analysis for the availability of C. Section and blood transfusion should be applied only for comprehensive EmONC facilities as you did it in table 3 and 4.

The application of QoC index and EmONC standard lacks integration. For example, a basic EmONC facilities providing all the seven signal functions should have a QoC index of 100%. But in your manuscript, it can be 77% (i.e seven out of the 9 signal functions).

On page 14 just before the caption for table 3, *"All 141 facilities in 2014 were asked if they had a nurse midwife or midwife and only 48% of these facilities had a midwife/ nurse"* my questions are 1) are you referring to a single nurse or midwife? You might need to give more contextual information, if any SPA surveyed health facilities can be run without a nurse or a midwife. Eg. Low level dispensaries may not need to have nurse/midwife. Taking this in mind your assessment should be focused on facilities providing EmONC services. 2) is there any health facility providing delivery are without having a midwife or a nurse to the least?

On page 15, just before the caption for table 5 "Analysis found a strong association between delivery rate and QoC index (p value 0.009), availability of trained provider (p value 0.004), total bed (p value 0.008) and the facility location (p value 0.097). We found that when QoC index increased by 0.01-unit, delivery rate increased by 143.61 delivery. Similarly, if facility has a provider trained on routine care for labor and delivery, delivery rate increased by 54.46 delivery and the association is statistically significant with a p value of 0.004" Here I see the chicken and the egg phenomenon. In the background/methods you have given evidence that facilities having more delivery have better quality of care. Hence is it the # of service provided which brings quality or is it the QoC which attracted more clients to come to the facility? which is difficult to answer using data from a cross sectional survey. Longitudinal design such as cohort could have been appropriate to address. Therefore, you should be careful in interpreting the findings, you can show association but not causal link/causality using the SPA and DHS data.

**Discussion section**

On the first paragraph of the discussion section, …" Only 22% facilities (31 out of 140) in 2014 and 23% facilities (32 in 141 facilities) in 2017 had cesarean delivery service". It is misleading to say only 22% /23% of the facilities in Bangladesh provide C-section. According to the EmONC standard only about one fourth/fifth of all health facilities are expected to provide C-section service, hence the prevalence should be calculated out of those supposedly providing the services. I would suggest you to re-run the analysis with the appropriate denominator

On page 16 paragraph 3 it says "The quality of delivery care was low both in 2014 and 2017 and remained unchanged between the two survey periods. Only 11 out of 135 facilities had all nine SF indicators at any point of time in 2017 compared to 8 facilities in 2014" It seems that you have used quality of delivery care to mean quality of EmONC by analyzing the availability of the nine-signal functions as predictor. I disagree as the two are different. In the analysis, you have included all the facilities irrespective of their EmONC status. But you should consider only the comprehensive EmONC facilities to assess whether they perform the nine signal functions.

**General comments**

The manuscript would benefit from language revision.

Here is a link for a paper on BEmONC status from Ethiopia that used primary and secondary data. https://bmcpregnancychildbirth.biomedcentral.com/articles/10.1186/1471-2393-14-354#Tab4
