# Supplementary material for: Association between ‘Emergency obstetric & newborn care readiness’ and delivery service utilization in Bangladesh: Evidence from national health facility assessment surveys
Source: PLoS One. 2025 May 27;20(5):e0297734. doi: 10.1371/journal.pone.0297734 (PMC12112276; doi:10.1371/journal.pone.0297734)
Supplement: Attachment — Submitted filename: Reviewer_comment_manuscript_by_Korotki_20240226.docx [file pone.0297734.s002.docx]

**Association between ‘Quality of Care’ and Delivery Service Utilization in Bangladesh: Evidence from National Health Facility Assessment Surveys**

Authors

Kaji Keya-Korotki, ((ORCID ID- 0000-0003-2396-4999)

Department: Health Services Policy and Management, University of South Carolina; Department of Health and Environmental Control, State of South Carolina (Current address)

Nabil Natafgi

Department: Health Services Policy and Management, University of South Carolina

Mark Macauda, Department: Health Promotion, Education, and Behavior, University of South Carolina

Syed Abdul Hamid, Department: Institute of Health Economics, University of Dhaka

M. Mahmud Khan, Department: Health Policy and management, University of Georgia

**Abstract**

This study assessed the Quality of Care (QoC) in the form of facility readiness, in terms of availability of human resources, equipment, and service availability in sub-district level Upazila Health Complex in Bangladesh in 2014 and 2017. Using regression models the study also assessed if QoC is associated with service utilization and readiness.

**Methods:** Using nationally representative Health Facility Assessment Survey and based on Donabedian model, quality was using a 9 indicators index signal function- antibiotics, oxytocin, anticonvulsant, blood transfusion, cesarean delivery, assisted vaginal delivery, manual removal of placenta, removal of retained product of conception and neonatal resuscitation. We ran multiple linear regression models using population standardized delivery rate and c-section delivery rate as the dependent variable.

**Results:** Caesarean section delivery was available in 31 facilities in 2014 and 32 facilities in 2017 whereas blood transfusion services in 31 facilities and 54 facilities respectively.

The availability of some signal function indicators increased between 2014 and 2017: oxytocic (85% to 95%), anticonvulsant (58% to 63%), and blood transfusion (22% to 38%). The average QoC index was 0.67 in 2014 and 0.69 in 2017 in terms of providing signal functions in the past 3 months. Only six facilities in 2014 and seven facilities in 2017 reported the ability to perform all the nine signal functions. Regression analysis found a strong association between delivery rate and QoC index (p-value 0.009).

**Conclusion:** Availability of several important readiness indicators increased: anticonvulsant, oxytocic, and blood transfusion system.

Although the mean QoC did not improve, it was due to the unavailability of C-section delivery care that depends on human resources, mainly gynecologist-anesthetist pair, supplies, lab facilities, technicians, and medicines.

Bangladesh should ensure each facility/hospital has an anesthetist-gynecologist pair available to provide care and make sure facilities have enough supplies and delivery care-related medicines to improve the QoC.

1. **Introduction**

Globally, maternal death reduction is a persistent challenge, and more than half of the maternal deaths result from hemorrhage, hypertensive disorders, and sepsis (Say et. Al., 2014). Some medical interventions termed `signal functions defined by the United Nations (UN) could be used for preventing those deaths (Paxton et al.,2005; WHO et al., 2009). A designated comprehensive emergency obstetric and newborn care (CEmONC) facility should have nine specific signal functions, such as (i) administering parenteral antibiotics, (ii) administering oxytocic or any uterotonic drugs for active management of the third stage of labor and prevention of postpartum hemorrhage, (iii) use of parenteral anticonvulsants for the management of pre-eclampsia/eclampsia, (iv) manual removal of placenta, (v) removal of retained products (e.g. manual vacuum extraction, dilatation, and curettage), (vi) performing assisted vaginal delivery (AVD), i.e. vacuum extraction or forceps delivery, (vii) performing basic neonatal resuscitation), (viii) performing CS delivery, and (ix) blood transfusion (BT) services to be available for 24 hours a day, 7 days a week (WHO et al., 2009; UNICEF, WHO, UNFPA, 1997; WHO, UNFPA, UNICEF, Mailman School of Public Health, 2009).

The maternal mortality ratio has decreased remarkably in many South Asian countries, including Bangladesh (WHO, OECD, & World Bank, 2018). While the country has reduced 40% of its maternal mortality ratio (MMR) in the last decades (NIPORT, MEASURE Evaluation & icddr,b, 2012), Bangladesh’s MMR rates remain higher than the neighboring countries (WB, 2017). Bangladesh adopted the policy of home delivery by a skilled provider in the last 20 years and that approach is helpful in reducing maternal deaths. Like most other developing countries in South Asia, the major causes of maternal deaths are hemorrhage and eclampsia/preeclampsia. These complications are essentially required to be managed at health facilities with relevant signal functions. For instance, hemorrhage can be compensated for blood transfusion; during administering parenteral oxytocin, manual removal of placenta, and removal of retained products, timely. BT may be necessary in the event of any hemorrhage. Administering parenteral anticonvulsants is essential for the management of eclampsia. Since these services are administered in health facilities, facility delivery is crucial for Bangladesh to further reduce the existing high level of maternal mortality.

Studies found associations between delivering in a health-care facility and survival of newborn babies in 67 low-income countries (Fink, Ross, & Hill, 2015; Bhutta et al., 2014). Many low-income countries encourage women to give birth at the nearest health facility that is designated to handle deliveries. Due to a lack of quality delivery care, maternal and neonatal deaths take place either in the facility or during transportation to better-equipped facility (Lama et al., 2020; Souza et al., 2013). Poor quality care is a continuous challenge to ensure in sub-Saharan Africa and south Asia, where both maternal and neonatal mortality is high and health systems are non-responsive (Kassebaum et al., 2014). Although researchers have identified serious quality deficits at some hospitals, the quality of care at lower-level facilities (example, health centers, and dispensaries) typically staffed with nurses or nursing aides have come under scrutiny (Hanson et al., 2013; Kruk et al., 2014; Chaturvedi et al., 2014; Hanson et al., 2015).

LMICs have research on the quality of obstetric care (Bintabara et al., 2019; Biswas et al., 2019; Kruk et al., 2016; Lama et al., 2020), but systematic research at lower-level facilities remains limited, partly because of the limited data availability (Hanson et al., 2013; Gabrysch et al., 2009; Nesbitt et al., 2013). The association between delivery rate and QoC is even more limited although the positive association between rate of deliveries and improved maternal and newborn baby outcomes was found in high-income countries. For example, US hospitals and providers with the lowest delivery rates less than 25 deliveries per month in hospitals, less than seven per year in health-care providers) had up to 50% more adverse maternal outcomes than hospitals and providers with higher delivery rates (Janakiraman et al., 2011; Kyser et al., 2012).

So far almost no systematic evidence is available either comparing the QoC using national survey or assessing the relation between QoC and delivery service uptake in Bangladesh although this country has one of the highest global burdens of maternal and newborn deaths. Kruk and colleagues found that more than 40% of facility deliveries in five African countries occurred in primary care facilities, which scored poorly on basic measures of maternal care quality, and facilities with caesarean section capacity, particularly those with birth rates higher than 500 per year, had higher scores for maternal care quality (Kruk et al., 2016). A similar study on the association between quality of care and delivery rate in Bangladeshi health facilities especially sub-district level health facilities are rare. To fill that gap, this study analyzed the quality of comprehensive maternal care functions as measured by signal function intervention in public health facilities in 2014 and 2017 and its association with rate of deliveries.

**1.1 Public health care structure in Bangladesh:** The country is divided into 8 divisions, 64 districts and 490 sub-districts. There are six types of public health facilities in Bangladesh: Community Clinic (CCs), Union Sub-Centers/Rural Dispensaries, union health and family welfare centers, upazila health complexes (UHCs), mother and child welfare centers (MCWCs), and district hospitals (DHs). Each district has a 100 beds district hospital, and each sub-district has a 31 or 50 beds sub-district health facility providing facility based EmONC care (MIS, 2018). District Hospitals (DH) and Maternal and Child Welfare Centers (MCWC) are situated at the district level, considered as urban and semi-urban area facilities, and works as referral center for UHCs or any other primary care centers. Sub-district level hospitals locally known as UHCs provides EmONC care in a sub-district and usually serves about 200,00-400,000 population. Below sub-district level, there is no facility-based delivery care. However, govt. of Bangladesh currently working to conduct normal delivery service in its union-level facilities.

2. **Method**

**2.1 Settings and type of facilities:** This manuscript studied the quality of delivery care of the sub-district facilities as they are the first level facility designated to provide facility-based delivery care. Government sanctions human resources, medicines, equipment, and all other arrangements to provide free EmONC care at these centers. A sub-district facility usually serves 200,000-400,00 population. In 2017, almost all of these UHCs were 50 beds facility i.e., designated to provide comprehensive EmONC care and rests 31 beds facility.

**2.2 Data:** Two datasets about childbirth and quality of care were used from service provision assessment (SPA) surveys, completed by the Bangladesh Demographic and Health Survey (DHS) program in 2014 and 2017. Population standardized delivery rate and service utilization data of 2017 were used to study the association of quality of delivery care and delivery service utilization rate.

**a. Service provision assessment survey:** The SPA survey is a cross-sectional survey conducted in district, sub-district, union, NGO and private facilities. The survey is conducted in every three years and due to COVID 19, the last available survey SPA 2017 was conducted in 2019. The datasets are available for public use from the DHS website (The DHS, USAID). In this article, we studied a total of 140 sub-district facilities surveyed in 2014 and 141 facilities surveyed in 2017. Brief description of both survey is available from the facility assessment report (NIPORT, ACPR, & ICF international, 2016; NIPORT & ICF, 2019).

**b. Service utilization dataset:** Delivery rate and service utilization data of the respected facilities was obtained from the yearly health bulletins published by the ministry of health and family welfare, Government of Bangladesh. The data is publicly available on the Directorate General of health Service’s website (MIS, 2017).

**2.3 Theoretical model:** This study is Based on Donabedian theoretical model of QoC and reflects upon structure, process, and outcome (Donabedian, 1988). Structure refers to personnel such as education, training, experience; Process indicator denotes activities taking place during diagnosis, prescription etc. Outcome indicator refers to the activity and quality of the process steps such as the rate of delivery, quality of life, disability, death, complications etc. Measures of structures and processes have useful aspects and can recognize specific weaknesses in the health systems that are actionable and are achievable to obtain in LMICs (Bilimoria, 2015).

**2.4 Study design and statistical analysis**

The study calculated the quality of the comprehensive emergency obstetric (nine signal function) index both for the availability of the care meaning if the facility ever performed that care, and also for the preparedness of the care meaning if the facility performed that care in the past 3 months.

Univariate frequency, bivariate cross tab, significance tests, and two multiple linear regression models were run. Besides univariate and bivariate analysis, mean, standard deviation, and proportion was calculated to find the readiness of the facilities along with multiple linear regression.

**2.5 Delivery service utilization:** The delivery rate was categorized into a few thresholds less than 52, between 53-183, 184-365, 366-500, and 501+ per year. These thresholds were selected for interpretability (52 is one delivery per week, 183 is one every other day, and 366 is one per day) and to reﬂect international thresholds (eg, 500 births per year) in the USA and UK, and provide a roughly balanced distribution of both facilities and births per category (Kruk et al., 2016).

**2.6 Regression model**

Multiple linear regression analysis was conducted to explore the association of population standardized delivery rate with QoC and some covariates. The dependent variable in the first model was the population-standardized delivery rate. A second model was run for the dependent variable- population standardized cesarean delivery rate.

$$Y_{t}\boldsymbol{=}\beta_{o}X_{0}+\beta_{1}X_{1}+\beta_{2}X_{2}+\beta_{3}X_{3}+\ldots+\beta_{k}X_{k}+u_{t}$$

𝑌_t_= dependent variable = population standardized delivery rate, population standardized cesarean delivery rate

𝑋_t_ = independent variables =SF index/ QoC index, trained provider, Kangaroo mother care etc.

**2.6 Dependent variable**

**Delivery rate:** Total delivery of each study facility was calculated by summing up all different kinds of delivery (number of normal, assisted, C-section deliveries) that took place in the study hospital in the year 2017. The number total delivery was converted into rates by dividing by the population of the sub-district and multiplying by 100,000.

**C-section delivery rate:** Total number of C-section delivery conducted at each UHCs.

The number of c-section delivery was converted into rates by dividing by the population of the sub-district and multiplying by 100,000.

**2.7 Independent variables**

**Signal function index/ qoc index:** QoC was measured as the availability of signal function indicators in the facility and the practice of signal function indicators in the past 3 months.

The mean or average of these 9 indicators was signal function quality score (QS). In signal function quality index, all 9 indicators are process indicators as these indicators takes place during diagnosis, prescription, and treatment. The index was compiled summing up all 9 available indexes and dividing by denominator 9.

For example, if a facility has 6 indicators available out of 9 indicators, the index was calculated as 6 divided by 9 making it 0.66. So, the index was a value between 0 to 1.

**Trained provider:** the facility has a provider who has received training on routine care for labor and delivery in past 2 years.

**Kangaroo mother care:** facility practice kangaroo mother care for low-birth-weight babies

**Guideline:** facility has a guideline either for conducting a normal delivery or caesarean delivery,

**Facility location- urban/ rural.** Although the study facilities are rural hospitals situated at the sub-district level, some UHCs are in semi-urban/ urban areas serving mostly the urban population from the catchment urban areas. Irrespective of their location, every UHCs covers a rural population, and this study will explore if the location or area makes a difference in the delivery service utilization.

**Partograph:** The facility uses a partograph to monitor labor and delivery.

**Availability of midwife/ nurse-midwife**: the facility has a midwife/ nurse-midwife to provide delivery service.

**Total beds:** Usually UHCs are either 31-beds or 50-bed hospitals. However, there were few 20 beds facilities and one facility had 100 beds.

3. **Result**

**3.1 Comparison of the service availability in 2014 and 2017:** There is no visible difference in the number of facilities that had been providing antenatal care, normal delivery, and cesarean delivery service between 2014 and 2017 survey periods. Table 1 shows that only 31 facilities in 2014 and 32 facilities in 2017 provided both normal and cesarean delivery. Facilities practicing kangaroo mother care increased from 78 to 88 between the two survey periods. Ninety-one facilities were reviewing maternal and neonatal deaths in 2014 while 78 facilities were doing the same in 2017.

***Insert Table 1 here.***

Service utilization data was publicly available since 2016 and included in this study for the year 2017. Out of 141 facilities in 2017, a total of 7 facilities had missing information and finally 134 facilities (141-7=134 facilities) were included in the service utilization analysis (Table 1). 29% percent facility had 53-183 delivery per year, another 29% facility had 184-365 delivery per year and only 26% facility had 500 plus delivery. Each facility on average had 588 admissions seeking maternal health care and conducted 385 delivery cases on average per year per facility. Each hospital had an average of 157 cases of intrapartum or delivery complications and 48 of those complications were referred to a different hospital.

***Insert Table 2 here.***

Table 2 shows each indicator of nine signal function availability at certain point of time in the facility. Total 135 facilities in 2014 and 136 facilities in 2017 offered normal delivery service and these facilities were asked during survey if they have performed/offered any of the 9 services (signal function indicators) to their clients. However, two of those indicators, availability of c-section delivery and blood transfusion system, were asked to all 140 facilities in 2014 and 141 facilities in 2017.

Analysis showed that availability of administering parental antibiotic was quite the same in 2014 and 2017. However, anticonvulsant service and oxytocic service increased. Assisted vaginal delivery service, another lifesaving service to treat obstructed delivery was available in 113 facilities in 2014 and reduced to 84 facilities in 2017.

The average or mean QoC score was 0.67 in 2014 and became 0.69 in 2017 (Table 2). Only a small number of facilities had all 9 lifesaving services available in 2014 and 2017. Analysis showed that 8 facilities in 2014 had all nine signal function indicators available compared to 11 facilities in 2017. Sixty-four facilities in 2014 had at least seven signal functions available while 75 offered the same service in 2017.

Figure 1 describes each of nine signal function availability in the facilities at least once in the past 3 months. Administering anticonvulsant saves a lot of life. In 2014, only 72 facilities had this service compared to 69 facilities in 2017. Similarly, 109 facilities had provided assisted vaginal delivery in 2014 compared to 80 facilities in 2017.

***Insert figure 1 here.***

Just 29 hospitals provided cesarean delivery service at least once in 2014 and 30 facilities provided that lifesaving treatment in 2017. No more than 6 facilities had all nine signal function indicators in 2014 and only 8 facilities had the same service in 2017. The mean or average QoC score was 0.62 in 2014 and became 0.64 in 2017.

**Infrastructure**

**3.2 Resource for providing delivery service.**

Thirty one facilities in 2014 and 32 facilities in 2017 were providing caesarean delivery care. Human resource availability of these facilities was studied, and analysis showed that 23 facilities in 2014 had a surgeon available or on call 24 hours to provide cesarean delivery compared to 25 facilities in 2017. All 23 facilities providing caesarean care in 2014 had a schedule of work or call list for that provider compared to 15 facilities out 25 facilities in 2017 had a call list in 2017. A surgeon and anesthetist, commonly known as pair is crucial for providing c-section delivery. In 2014, 22 facilities had a pair compared to 18 facilities in 2017. All 141 facilities in 2014 were asked if they had a nurse midwife or midwife and only 48% of these facilities had a midwife/ nurse.

***Insert Table 3 here.***

**3.3 Functional caesarean delivery equipment**

Table 4 shows the list of caesarean delivery equipment available and functional in 2014 and 2017 among the facilities that provide cesarean delivery service. Only 27 facilities had a functional anesthesia machine in 2014 compared to 28 facilities in 2017. All 31 facilities in 2014 and 32 facilities in 2017 had operation table, light and intra vine stand.

***Insert Table 4 here.***

**3.4 Empirical models for association**

Table 5 shows the association of population delivery rate and independent variables—QoC index, available provider trained on routine care for labor and delivery in past 2 years, practicing kangaroo mother care, number of total beds, facility has guideline for conducting normal or c-section delivery, facility has a midwife or nurse midwife, facility location and use of partograph (Model 1). This analysis included a total of 127 facilities out of 140 facilities from the 2017 survey as rest of the facilities either did not provide delivery care or had not had the delivery rate information. Analysis found a strong association between delivery rate and QoC index (p value 0.009), availability of trained provider (p value 0.004), total bed (p value 0.008) and the facility location (p value 0.097). We found that when QoC index increased by 0.01-unit, delivery rate increased by 143.61 delivery. Similarly, if facility has a provider trained on routine care for labor and delivery, delivery rate increased by 54.46 delivery and the association is statistically significant with a p value of 0.004.

***Insert Table 5 here.***

A strong and statistically significant association was also found between number of c-section delivery and QoC index (p value 0.003), total bed (p value 0.071) and the facility location (p value 0.03). The association between standardized c-section delivery rate is shown in model 2

4. **Discussion and conclusion**

Using nationally representative data from health facility surveys in Bangladesh, we found that nearly 96% facilities (135 in 140) in 2014 and 97% facilities (136 in 141) in 2017 provided normal delivery care. Only 22% facilities (31 out of 140) in 2014 and 23% facilities (32 in 141 facilities) in 2017 had cesarean delivery service. Seventy eight percent of facilities in 2014 and 77% facilities in 2017 providing delivery care services did not have the capacity to provide cesarean delivery care. Seventy four percent facilities in 2014 and 73% facilities in 2017 had blood grouping and testing facilities.

Overall, 63% facilities (36 in 134 facilities) delivered fewer than 500 deliveries per year. Delivery in high-income countries usually takes place in well-equipped facilities with exceptions for remote locations or birth centers. Studies showed that only 2% of births per year in Finland, less than 5% of births in England, and less than 8% of births per year in the USA occurred in health care facilities fewer than 500 deliveries per year (Pyykonen et al., 2014; Redshaw et al., 2011; Simpson KR, 2011).

Availability of several important readiness indicators increased between the two survey periods such as anticonvulsant, oxytocic, and blood transfusion system. The availability of five, seven and 9 signal functions also increased between the two-survey period.

The quality of delivery care was low both in 2014 and 2017 and remained unchanged between the two survey periods. Only 11 out of 135 facilities had all nine SF indicators at any point of time in 2017 compared to 8 facilities in 2014. A study conducted in Bangladesh showed that SF indicators were available in district hospitals and maternal and child welfare centers but absent in sub-district hospitals (Roy et al., 2017). The findings of less availability of signal functions are consistent with the previous study conducted in public and private hospitals in Bangladesh (Alam et al., 2015).

Findings showed that the overall mean quality score was 0.67 in 2014 among the facilities that offered delivery care and only became 0.69 in 2017. The analysis also showed that the SF availability (QoC index) at least once in the past 3 months of the survey was 0.62 in 2014 and became 0.64 in 2017. These findings are similar to individual studies showing weak infrastructure, staffing, and emergency care capacity in low-income countries (Lama et al., 2020; Kruk et al., 2016; Hsia et al., 2012; Baily et al., 2006; Hongoro & McPake, 2004; Anand & Bärnighausen, 2004).

No apparent change in the quality between the two surveys could be the possible reason why maternal death did not decrease. Bangladesh still has high maternal mortality and only 65% of facilities (91 out of 135) in 2014 and 57% of facilities (78 out of 136) in 2017 regularly reviewed maternal or neonatal deaths or near-missed cases. On average, 391 delivery care was provided per facility per year arising 162 complications and each facility referred 48 complicated cases in 2017. Maternal death at the facility was low (0.13 death per facility) but stillbirth, neonatal death, and near missed cases were high in number. This big burden of complications, referrals, stillbirths, and neonatal deaths could have been avoided with a robust infrastructure and competent and alert staff. The emergency referral is a common practice for community-level or lower-level facilities to deal with complications. However, long distances to next-level facilities, road conditions, transport fares, and slow recognition of the severity of the complications by the client’s attendants are barriers to reach a referral hospital resulting maternal band newborns death during referral (Hirose et al., 2015; Assarag et al., 2015).

We noted a strong association between obstetric care quality index and delivery rate. Additional factors associated with delivery rate are provider having training on routine care for labor and delivery in past 2 years, total bed number, and facility location i.e. urban-rural. Kruk and colleagues found a strong association between QoC index and the co variates- delivery rate, number of skilled staff per bed, facility type i.e. public vs private etc.

We had several limitations. First, SPA data is collected using a cross sectional survey method, not a longitudinal survey method. So, the comparison between 2014 and 2017 does not exactly reflect the quality changes among the same exact facilities. Secondly, we had not had the utilization/outcome data (number of deliveries, complications, referrals etc.) for the year of 2014 and therefore we could not compare outcome data in two different survey periods. We had data on staffing at health-care facilities, but we do not have data for provider skill or competence in obstetric care especially. We also could not measure the respectful treatment during delivery as this element was out of our scope. Disrespectful behavior and abuse are common and might discourage women from seeking care. Future studies might bring a more comprehensive quality assessment and bring important insights; our index provides a checklist to proof if facilities possess cesarean delivery and complication management capacity.

To the best of our knowledge, this is the first study using nationally representative health system data and combined with outcome/ utilization data to systematically assess the comprehensive obstetric care quality in two different time points. Further studies are needed to build on this research. Determinants of quality need to be investigated more.

Our study brings important findings and add in the scientific literature of quality of care identifying gaps in provision of comprehensive obstetric care. Quality improve is a persistent challenge in most LMICs. Countries take various policies to improve utilization and quality. For example, India took ‘Janani Suraksha Yojana’ program that provides a cash incentive for women to deliver in facilities (Lim et al., 2010). The JSY program increased facility delivery by 50% in the first year and to some extent reduced the neonatal mortality, but no effect was found on maternal mortality (Lim et al., 2010; Randive, Diwan & Costa, 2013; Paul, 2010). Bangladesh introduced a similar program- ‘Demand Side Finance Voucher Program’ in the hard-to-reach rural areas. The available evaluation found that Bangladesh’s voucher program did not improve quality (Talukder et al., 2014).

Many lower income countries had experimented with promoting delivery in higher rate settings (Moran, Naidoo & Moodley, 2010; Hofmeyr et al., 2014). However, this approach means that the number of facilities with capability to do caesarean sections might also need to increase and might not be feasible for Bangladesh in a short period of time. To meet the Sustainable Development Goals, Bangladesh must carefully choose the policy and implement effectively to meet its obligations to the population to provide safe delivery care services.

**8. References**

1.	Say L, Chou D, Gemmill A, Tuncalp O, Moller AB, Daniels J et al. Global causes of maternal death: a WHO systematic analysis. Lancet Glob Health. 2014 Jun; 2(6):e323±33. https://doi.org/10.1016/S2214-109X(14)70227-X PMID: 25103301

2.	Paxton A, Maine D, Freedman L, Fry D, Lobis S. The evidence for emergency obstetric care. Int J GynecolObstet. 2005; 88:181±193.

3.	World Health Organization, United Nations Population Fund, United Nations Children's Fund, Averting Maternal Death and Disability Program. Monitoring Emergency Obstetric Care: A Handbook. Published 2009 <http://www.unfpa.org/sites/default/files/pub-pdf/obstetric_monitoring.pdf>.

4.	United Nations Children's Fund, World Health Organization, United Nations Population Fund. 1997. Guidelines for Monitoring the Availability and Use of Obstetric Services. https://www.unicef.org/health/files/guidelinesformonitoringavailabilityofemoc.pdf .

5.	WHO, UNICEF, UNFPA, World Bank Group, and the United Nations Population Division. Trends in Maternal Mortality: 1990 to 2015. Geneva, World Health Organization, 2015. <http://data.worldbank.org/indicator/SH.STA.MMRT?view=chart>.

6.	National Institute of Population Research and Training (NIPORT), MEASURE Evaluation, icddr,b. Bangladesh Maternal Mortality and Health Care Survey 2010. 2012. http://www.cpc.unc.edu/measure/publications/tr-12-87.

7.	World Health Organization, Organization for Economic Co-operation and Development, and The World Bank. Delivering quality health services: a global imperative for universal health coverage. Geneva, 2018. https://extranet.who.int/sph/docs/file/1654

8.	Souza JP, Gülmezoglu AM, Vogel J, Carroli G, Lumbiganon P, Qureshi Z, et al. Moving beyond essential interventions for reduction of maternal mortality (the WHO Multicountry Survey on Maternal and Newborn Health):a cross-sectional study. Lancet. 2013 18;381(9879):1747–1755.

9.	Pyykonen A, Gissler M, Jakobsson M, Petäjä J, Tapper AM. Determining obstetric patient safety indicators: the diff erences in neonatal outcome measures between diff erent-sized delivery units. BJOG 2014; 121: 430–37.

10.	Redshaw M, Rowe R, Schroeder L, et al. Mapping maternity care: the confi guration of maternity care in England. Birthplace in England research programme. Final report part 3. Southampton; NIHR Service Delivery and Organisation programme, 2011.

11.	Simpson KR. An overview of distribution of births in United States hospitals in 2008 with implications for small rate perinatal units in rural hospitals. J Obstet Gynecol Neonatal Nurs 2011; 40: 432–29.

12.	Hsia RY, Mbembati NA, Macfarlane S, Kruk ME. Access to emergency and surgical care in sub-Saharan Africa: the infrastructure gap. Health Policy Plan 2012; 27: 234–44.

13.	Hongoro C, McPake B. How to bridge the gap in human resources for health. Lancet 2004; 364: 1451–56.

14.	Anand S, Bärnighausen T. Human resources and health outcomes: cross-country econometric study. Lancet 2004; 364: 1603–09.

15.	Bailey P, Paxton A, Lobis S, Fry D. The availability of life-saving obstetric services in developing countries: an in-depth look at the signal functions for emergency obstetric care. Int J Gynecol Obstet 2006; 93: 285–91

16.	Kruk ME, Leslie HH, Verguet S, Mbaruku GM, Adanu RMK, Langer A. Quality of basic maternal care functions in health facilities of five African countries: an analysis of national health system surveys. Lancet Glob Health. 2016 Nov;4(11):e845-e855. doi: 10.1016/S2214-109X(16)30180-2. Epub 2016 Sep 23. PMID: 27670090.

17.	Lama TP, Munos MK, Katz J, Khatry SK, LeClerq SC, Mullany LC. Assessment of facility and health worker readiness to provide quality antenatal, intrapartum and postpartum care in rural Southern Nepal. BMC Health Serv Res. 2020 Jan 6;20(1):16. doi: 10.1186/s12913-019-4871-x. PMID: 31906938; PMCID: PMC6945781.

18.	Hirose A, Borchert M, Cox J, Alkozai A, Filippi V. Determinants of delays in travelling to an emergency obstetric care facility in Herat, Afghanistan: an analysis of cross-sectional survey data and spatial modelling. BMC Pregnancy Childbirth 2015; 15: 14.

19.	Assarag B, Dujardin B, Delamou A, Meski FZ, De Brouwere V. Determinants of maternal near-miss in morocco: too late, too far, too sloppy? PLoS One 2015; 10: e0116675.

20.	Lim SS, Dandona L, Hoisington JA, James SL, Hogan MC, Gakidou E. India’s Janani Suraksha Yojana, a conditional cash transfer programme to increase births in health facilities: an impact evaluation. Lancet 2010; 375: 2009–23.

21.	Randive B, Diwan V, De Costa A. India’s conditional cash transfer programme (the JSY) to promote institutional birth: is there an association between institutional birth proportion and maternal mortality? PLoS One 2013; 8: e67452.

22.	Paul VK. India: conditional cash transfers for in-facility deliveries. Lancet 2010; 375: 1943–44

23.	Moran NF, Naidoo M, Moodley J. Reducing maternal mortality on a countrywide scale: the role of emergency obstetric training. Best Pract Res Clin Obstet Gynaecol 2015; 29: 1102–18.

24.	Hofmeyr GJ, Mancotywa T, Silwana-Kwadjo N, Mgudlwa B, Lawrie TA, Gulmezoglu AM. Audit of a new model of birth care for women with low risk pregnancies in South Africa: the primary care onsite midwife-led birth unit (OMBU). BMC Pregnancy Childbirth 2014; 14: 417.

25.	Hanson C, Ronsmans C, Penfold S, et al. Health system support for childbirth care in Southern Tanzania: results from a health facility census. BMC Res Notes 2013; 6: 435

26.	Hanson C, Cox J, Mbaruku G, et al. Maternal mortality and distance to facility-based obstetric care in rural southern Tanzania: a secondary analysis of cross-sectional census data in 226 000 households. Lancet Glob Health 2015; 3: e387–95.

27.	Janakiraman V, Lazar J, Joynt KE, Jha AK. Hospital rate, provider rate, and complications after childbirth in US hospitals. Obstet Gynecol 2011; 118: 521–27.

28.	Kyser KL, Lu X, Santillan DA, et al. The association between hospital obstetrical rate and maternal postpartum complications. Am J Obstet Gynecol 2012; 207: 42 e1–17.

29.	The Demographic and Health Surveys. The DHS program. https://www.dhsprogram.com/data/Using-DataSets-for-Analysis.cfm

30.	Management Information System (MIS). 2017. Local Health Bulletin. Directorate General of Health Services (DGHS). Ministry of Health & Family Welfare. People’s Republic of Bangladesh. <http://103.247.238.81/webportal/pages/rtlhb_menu.php>

31.	World Bank (WB) Data. <https://data.worldbank.org/>

32.	National Institute of Population Research and Training (NIPORT) and ICF. 2019. Bangladesh Health Facility Survey 2017. Dhaka, Bangladesh: NIPORT, ACPR, and ICF.

33.	Management Information System (MIS). 2018. Health Bulletin 2018. Directorate General of Health Services. Ministry of Health and Family Welfare. Government of the People’s Republic of Bangladesh.

34.	Bintabara D, Ernest A, Mpondo B. Health facility service availability and readiness to provide basic emergency obstetric and newborn care in a low-resource setting: evidence from a Tanzania National Survey. BMJ Open 2019;9:e020608. doi:10.1136/ bmjopen-2017-020608

35.	Biswas TK, Sujon H, Rahman MH, Perry HB, Chowdhury ME. Quality of maternal and newborn healthcare services in two public hospitals of Bangladesh: identifying gaps and provisions for improvement. BMC Pregnancy Childbirth. 2019 Dec 10;19(1):488. doi: 10.1186/s12884-019-2656-1. PMID: 31823747; PMCID: PMC6905111.

36.	Shakil A, Khan M. A maternal health voucher scheme: what have we learned from the demand-side financing scheme in Bangladesh?, Health Policy and Planning, Rate 26, Issue 1, January 2011, Pages 25–32, https://doi.org/10.1093/heapol/czq015

37.	Talukder MN, Rob U, Musa SAJM, Bajracharya A, Keya KT, Noor FR, Jahan E, Hossain MI, Saha J, Bellows B. 2014. "Evaluation of the impact of the voucher program for improving maternal health behavior and status in Bangladesh," Final report. Dhaka: Population Council.

38.	Fink G, Ross R, Hill K. Institutional deliveries weakly associated with improved neonatal survival in developing countries: evidence from 192 Demographic and Health Surveys. Int J Epidemiol 2015; published online July 30. DOI:10.1093/ije/dyv115.

39.	Bhutta ZA, Das JK, Bahl R, et al. Can available interventions end preventable deaths in mothers, newborn babies, and stillbirths, and at what cost? Lancet 2014; 384: 347–70.

40.	Kassebaum NJ, Bertozzi-Villa A, Coggeshall MS, et al. Global, regional, and national levels and causes of maternal mortality during 1990–2013: a systematic analysis for the Global Burden of Disease Study 2013. Lancet 2014; 384: 980–1004

41.	Chaturvedi S, Randive B, Diwan V, De Costa A. Quality of obstetric referral services in India’s JSY cash transfer programme for institutional births: a study from Madhya Pradesh province. PLoS One 2014; 9: e96773

42.	Nesbitt RC, Lohela TJ, Manu A, et al. Quality along the continuum: a health facility assessment of intrapartum and postnatal care in Ghana. PLoS One 2013; 8: e81–89.

43.	Donabedian A. The quality of care. How can it be assessed? JAMA. 1988;260(12):1743‐1748. doi:10.1001/jama.260.12.1743

44.	Bilimoria KY. Facilitating quality improvement: pushing the pendulum back toward process measures. JAMA 2015; 314: 1333–34

45.	Roy L, Biswas TK, Chowdhury ME (2017) Emergency obstetric and newborn care signal functions in public and private facilities in Bangladesh. PLoS ONE 12(11): e0187238. <https://doi.org/10.1371/journal.pone.0187238>

46.	Alam B, Mridha MK, Biswas TK, Roy L, Rahman M, Chowdhury ME. Coverage of emergency obstetric care and availability of services in public and private health facilities in Bangladesh. Int J Gynaecol Obstet. 2015; 131:63–9. https://doi.org/10.1016/j.ijgo.2015.04.041 PMID: 26165908
